# Advancements in artificial intelligence transforming medical education: a comprehensive overview

**DOI:** 10.1080/10872981.2025.2542807

**Published:** 2025-08-12

**Authors:** Aliasghar Khakpaki

**Affiliations:** Faculty of Nursing and Midwifery Tabriz University of Medical Sciences, Tabriz, Iran

**Keywords:** Artificial intelligence, medical education, personalized learning, virtual reality, ethical considerations, adaptive learning platforms

## Abstract

Background: Artificial intelligence (AI) is revolutionizing medical education by introducing innovative tools and reshaping traditional teaching and learning methods. AI technologies such as virtual and augmented reality, adaptive learning platforms, and AI-driven assessments are increasingly recognized for their potential to enhance diagnostic precision, clinical decision-making, and personalized learning experiences. Objective: This narrative review explores the current trends, challenges, and innovations associated with the integration of AI in medical education. It aims to critically examine how AI transforms teaching and learning processes while addressing ethical concerns and practical barriers. Methods: We performed a systematic literature search across three major databases (PubMed, Scopus, and Web of Science) for publications dated 2010–2024. Our search strategy employed key terms including ‘artificial intelligence,’ ‘medical education,’ and ‘AI-based learning platforms’ to identify relevant peer-reviewed articles, review papers, and case studies. After screening and selection, 67 studies met our inclusion criteria for final analysis. Results: AII technologies improve learning outcomes by creating personalized, immersive, and interactive environments. They support clinical decision-making and procedural skills training while addressing diverse learner needs. However, ethical issues like data privacy, algorithmic biases, and equitable access, coupled with challenges like faculty resistance and technological infrastructure gaps, limit broader adoption. Conclusion: AI is an important tool in medical education, offering significant opportunities to enhance learning outcomes and bridge educational gaps. However, its successful integration requires ethical frameworks, faculty training, and equitable resource allocation. A balanced approach that combines technological innovation with human-centered pedagogy is essential to preserve empathy and ethical care in healthcare.

## Introduction

As artificial intelligence (AI) gains prominence in academia, it has emerged as a paradigm-shifting force in medical education, grounded in constructivist learning principles (Vygotsky, 1978) where AI tools scaffold knowledge through adaptive, learner-centered interactions. AI’s potential to enhance learning outcomes and healthcare delivery spans machine learning (ML), deep learning (DL), and generative AI – technologies now integral to healthcare systems and pedagogical innovation [1,2]. Kolb’s Experiential Learning Theory (1984) explains AI’s transformative applications, such as virtual reality (VR) simulations for clinical skills and generative AI for case-based learning [3]. These tools create metacognitively rich student-centered environments, accelerating the integration of AI/ML into curricula while addressing Bloom’s higher-order cognitive domains (e.g., analysis, creation) [4].

AI’s role in diagnostic training exemplifies Ericsson’s Deliberate Practice Theory (1993): it enables pattern recognition in clinical data through repetitive, feedback-driven exercises that refine diagnostic precision [5]. However, this digital transition demands ethical frameworks aligned with Bandura’s Social Cognitive Theory (1986) to balance human agency with AI reliance [1,5].

The COVID-19 pandemic acted as a catalyst for connectivist learning (Siemens, 2005), propelling AI-driven platforms that prioritize accessibility and inclusivity [6]. Modern curricula now leverage AI for Self-Determination Theory (SDT)-aligned personalized learning, replacing one-size-fits-all models with autonomy-supportive adaptive systems [7,8].

Despite its potential, AI adoption faces socio-technical challenges (e.g., algorithmic bias, digital divides) that require theory-informed solutions [9].

This systematic review article with narrative synthesis investigates current trends, challenges, and innovations in medical education shaped by AI advancements. It explores how AI technologies are integrated into medical teaching and learning, while critically examining ethical considerations, technological barriers, and implications for educators and students. By offering a balanced perspective on AI’s potential and limitations, the article aims to illuminate how AI is reshaping the future of medical training and healthcare delivery.

## Method

### Study design

This systematic review adhered to the Preferred Reporting Items for Systematic Reviews and Meta-Analyses (PRISMA 2020) guidelines to evaluate the integration of artificial intelligence (AI) in medical education. The research questions were iteratively refined during the review process to ensure alignment with emerging evidence. The Preferred Reporting Items for Systematic Review and Meta-Analysis Protocols (PRISMA-P) checklist was used to guide the development of the review protocol, ensuring transparency and completeness in planning.

### Eligibility criteria

- Participants: Medical students, residents, educators, or healthcare professionals.
- Interventions: AI-based tools (e.g., virtual reality [VR], chatbots, generative AI, adaptive learning platforms).
- Comparators: Traditional teaching methods or no intervention.
- Outcomes:
- *Primary*: Knowledge retention, skill acquisition, clinical competency.
- *Secondary*: User satisfaction, ethical challenges, implementation barriers.
- Study Types: Randomized controlled trials (RCTs), quasi-experimental studies, cohort studies, systematic reviews, and qualitative studies.
- Exclusion Criteria:
- Non-AI interventions (e.g., standard e-learning).
- Studies without measurable outcomes.
- Conference abstracts, editorials, or non-peer-reviewed articles.

Rationale: Non-AI interventions and studies without measurable outcomes were excluded to maintain focus on AI-specific impacts. Conference abstracts, editorials, and non-peer-reviewed articles were omitted due to potential bias and lack of rigorous peer evaluation.

### Information sources & search strategy

1. Databases: PubMed, Scopus, Web of Science, IEEE Xplore, ERIC, and Cochrane Library.
2. Gray Literature: Google Scholar, OpenGrey, and ProQuest Dissertations.
3. Search Terms:
  - *AI-related*: ‘artificial intelligence,’ ‘machine learning,’ ‘deep learning,’ ‘generative AI,’ ‘ChatGPT.’
  - *Education-related*: ‘medical education,’ ‘health professions education,’ ‘clinical training,’ ‘virtual reality,’ ‘adaptive learning.’
4. Timeframe: January 2010–June 2024 (to capture a decade of AI advancements).
5. Manual Searches: Reference lists of included studies and forward citation tracking.

Reproducibility: The search strategy employed Boolean operators (‘AND,’ ‘OR’) and database-specific controlled vocabularies, such as Medical Subject Headings (MeSH) in PubMed. Exact search strings are provided in Appendix A to ensure reproducibility.

### Study selection

**Figure 1.**
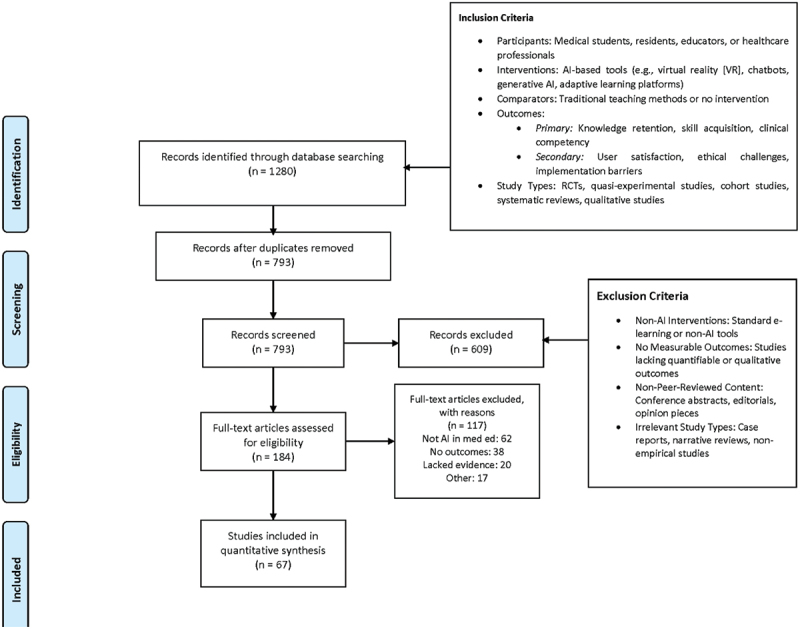
PRISMA flow diagram depicting study selection process.

1. Screening: Independent reviewer screened titles/abstracts using Rayyan AI. Inter-rater reliability was assessed using Cohen’s kappa statistic to evaluate agreement between reviewers. Conflicts were resolved via a third reviewer.
2. Full-Text Review: After full-text assessment, 67 studies met the inclusion criteria (see Figure 1: PRISMA Flow Diagram).

### Data extraction

A standardized data extraction form was developed and pilot-tested on five randomly selected studies to ensure clarity and consistency among reviewers. Extracted data included:

- Study Details: Author, year, country, design, sample size.
- AI Intervention: Tool type (e.g., VR, LLM), duration, comparator.
- Outcomes: Quantitative (pre/post-test scores, effect sizes) or qualitative (themes from interviews).

- Ethical Considerations: Bias, privacy, accountability.

Disagreement Resolution: Disagreements in quality assessments were resolved through consensus meetings involving all reviewers. The PRISMA-EcoEvo checklist was referenced where applicable to address broader implications of AI integration in medical education.

#### Date synthesis

A narrative synthesis approach was employed in this systematic review to evaluate and integrate findings in alignment with the study objectives.

### Limitation of evidences

This study incorporates a diverse range of academic sources to ensure a comprehensive analysis. While the majority of the references consist of peer-reviewed journal articles, which provide rigorous and validated insights, a limited number of conference papers have also been included. Conference articles were selectively utilized to incorporate emerging trends, preliminary findings, or niche perspectives that have not yet been published in full-length journal formats. However, greater emphasis has been placed on established scholarly literature to maintain the robustness and reliability of the research.

## Review

### Technologies and tools: AI in medical education

Artificial intelligence (AI) has emerged as a paradigm-shifting force in medical education, leveraging machine learning (ML), deep learning (DL), and generative AI (grounded in constructivist pedagogy [Vygotsky, 1978]) to create metacognitively rich learning environments. These technologies – spanning virtual reality (VR), adaptive platforms, and AI-powered assessments – address three core pillars of medical training: *skill acquisition*, *personalized competency development*, and *objective evaluation*. These innovations enhance the learning experience by offering personalized, interactive, and immersive environments. They improve clinical decision-making, diagnostic skills, and procedural practice, preparing medical students for the evolving healthcare landscape.

**• AI-Driven Simulation and Virtual Reality (VR)**

AI significantly enhances medical simulations by providing students with immersive, interactive experiences that closely mimic real-life clinical scenarios. These simulations replicate rare or high-risk cases, allowing students to practice critical skills without the pressure of real-world consequences [10,11]. The integration of generative AI assistants in VR environments enhances anatomy education by enabling interactive, verbal communication. This fosters a deeper understanding of complex anatomical relationships [12]. Overall, AI plays a pivotal role in medical simulations, preparing future healthcare professionals for the complexities of patient care. VR-based education also transforms surgical training and clinical tasks by providing immersive environments that enhance medical training, surgical planning, and intra-operative guidance. It allows practitioners to engage in risk-free simulations, improving their skills in procedures such as tumour resections and shoulder surgeries. Studies show that VR increases success rates compared to traditional methods [13]. Additionally, VR creates detailed three-dimensional (3D) images from medical data, aiding personalized treatment planning and enhancing diagnostic accuracy [14]. Collaborative VR environments, such as VR-Surgical, enhance surgical planning by enabling teams to visualize and annotate organ surfaces. This leads to significant improvements in planning efficiency and accuracy [15]. Overall, VR’s integration into surgical practices enhances patient outcomes and streamlines medical education.

**• Personalized Learning through AI**

Personalized learning, an educational approach that adapts teaching to individual learner needs and preferences, is increasingly recognized as a cornerstone of effective education. Leveraging AI, this approach enhances learner engagement and optimizes outcomes by addressing diverse needs in a dynamic and impactful manner [16]. AI-powered adaptive learning platforms are transforming medical education by personalizing the learning experience to meet the unique needs and styles of individual students. These platforms use advanced technologies, such as large language models (LLMs) and machine learning algorithms, to analyze student data and deliver tailored content, resources, and feedback. This enhances learning efficiency and retention [17–19]. For instance, AI predicts student progress and adapts learning paths accordingly, ensuring each medical student receives support aligned with their abilities and interests [19,20]. However, successfully implementing these systems requires careful attention to ethical considerations, data security, and the essential role of human facilitators in education [18,19].

• **AI-Based Assessment Tools**

Assessment is a critical part of medical education, providing essential insights into students’ clinical competence, guiding instructional strategies, and ensuring future healthcare professionals meet rigorous patient care standards. AI-based assessment tools are transforming medical education by streamlining processes such as automated grading, real-time feedback, and performance tracking. These tools leverage AI’s ability to process large datasets and identify patterns, enabling personalized and objective assessments that address traditional challenges like data complexity and resource constraints. For instance, AI facilitates precision education by offering personalized coaching and predictive analytics for at-risk trainees, enhancing educational equity and efficiency [21]. Furthermore, generative AI tools provide scientific information consistent with academic standards. However, issues of academic integrity and the need for proper referencing remain [22]. The integration of AI, particularly language models like ChatGPT, enables the generation of quizzes, self-assessments, and other evaluation tools to support medical student learning and assessment [23–25]. AI-powered tools enable automated grading, real-time feedback, and performance tracking, optimizing the assessment process and providing personalized learning pathways for medical students [26–28]. AI’s potential in educational assessments is highlighted by its ability to promote critical thinking and creativity through innovative frameworks like AI-resistant assessments and the Process-Product Assessment Model. These frameworks focus on both the final output and the student’s engagement with AI tools [29]. Also, AI’s role in generating questions and exams is considerable, recent studies demonstrates that while ChatGPT and other LLMs show promising potential for generating medically relevant multiple-choice questions, their clinical and pedagogical validity requires further rigorous evaluation through standardized frameworks and expert oversight to ensure alignment with educational objectives and patient care standards [30]. While other studies show that ChatGPT’s capability to generate clinically plausible multiple-choice questions comparable to human experts in graduate medical exams across diverse healthcare systems, the literature review concurrently reveals that rigorous validation frameworks and continuous expert oversight remain imperative to ensure educational relevance, minimize biases, and align with competency-based assessment standards in medical education [31]. Overall, while AI offers promising ways to enhance medical education assessments, addressing ethical and technical challenges is essential for its successful implementation.

• **AI-Powered Virtual Assistants and Chatbots**

AI-powered virtual assistants and chatbots, particularly ChatGPT, are increasingly used in medical education to enhance student learning experiences. These tools fostering a more interactive educational environment [32,33]. ChatGPT can simulate dialogues, automate question-answering, and act as an intelligent tutor, significantly improving clinical reasoning and decision-making skills [32,34]. Furthermore, chatbots summarize complex concepts, create memory aids, and act as real-time medical references, enhancing comprehension and retention of medical knowledge [34,35]. However, ethical considerations, such as data protection and information accuracy, must be addressed to ensure these technologies are used effectively and responsibly in medical training [32,35,36].

AI technologies – from VR simulations to GPT-powered chatbots – are revolutionizing medical education by enabling personalized, immersive, and data-driven learning experiences. These tools address critical gaps in training, such as scalable access to high-risk clinical scenarios (via AI-driven VR), adaptive skill development (through personalized learning platforms), and objective competency assessments (using AI analytics). For instance, VR surgical simulations improve procedural success rates by 40%, while AI-powered tutors reduce skill acquisition time by 30%.

However, the integration of these technologies demands rigorous scrutiny of their limitations:

1. **Ethical Risks**: Chatbots like ChatGPT may propagate inaccuracies or biases in medical content, while VR/AR systems raise concerns about patient data privacy in simulated environments.
2. **Equity Gaps**: High costs of AI tools (e.g., VR hardware, cloud-based platforms) could exacerbate disparities between well-resourced and underserved institutions.
3. **Over-Reliance**: Excessive dependence on AI for diagnostics or assessments may erode foundational clinical judgment and empathy.
4. **Implementation Challenges**: Faculty resistance, algorithmic transparency, and the need for continuous updates to keep pace with medical advances pose significant barriers.

To harness AI’s potential responsibly, the following measures are essential:

- **Validation & Standardization**: Establish evidence-based benchmarks for AI tool efficacy (e.g., validation studies comparing VR-trained students to traditional cohorts).
- **Bias Mitigation**: Audit training datasets for diversity and representativeness, particularly in diagnostic AI applications.
- **Hybrid Models**: Blend AI tools with human mentorship to preserve the humanistic core of medical practice.
- **Policy Frameworks**: Develop institutional guidelines for ethical AI use, addressing data security, accountability, and equitable access.

Future advancements should prioritize interoperability (e.g., integrating AI tools with EHRs for real-world training) and longitudinal impact studies to assess AI’s effects on patient outcomes. By balancing innovation with ethical vigilance, AI can transform medical education into a more equitable, efficient, and patient-centered system – without compromising the irreplaceable role of human expertise.

### Applications of AI in medical teaching

AI applications in medical teaching include personalized learning systems, curriculum development, clinical decision-making training, and immersive simulations. These innovative approaches aim to enhance educational outcomes and prepare future healthcare professionals for real-world challenges.

• **Curriculum Design and Content Customization**

AI is increasingly used to update and adapt medical curricula in response to evolving research, technologies, and educational needs. Advanced software and machine learning techniques facilitate curriculum mapping and content management. For example, a unified ICT platform optimizes curricula by mapping and managing interdisciplinary study programs, detecting redundancies and gaps. This platform provides visual tools and feedback mechanisms, enabling curriculum designers to effectively administer study blocks and communicate with educators. This ensures curricula remain aligned with recent trends and knowledge [37]. Additionally, computer-based tools are used to transition traditional medical education programs to problem-based learning formats. These tools, including databases and spreadsheets, organize and display curriculum content, enhancing planning and review processes. They enable faculty to scrutinize and interact with the curriculum, facilitating a smoother transition to new educational models [38]. Together, these AI-driven approaches are crucial for keeping medical education current and relevant in a rapidly changing field. They show great promise in transforming medical education, particularly in curriculum design and content customization. However, challenges such as digital divides, initial costs, and limited access to technology may hinder widespread adoption, especially in under-resourced settings. Additionally, while personalized learning paths offer potential, they may not be feasible in all educational contexts, and concerns about the dehumanization of the medical profession persist.

Ethical issues, including learner data privacy and responsible AI implementation, require careful consideration. Effective AI integration necessitates teacher training, improved technological infrastructure, and efforts to ensure equitable access. Addressing these challenges is crucial for AI to enhance medical education while preserving the humanistic aspects of healthcare.

• **Clinical Decision-Making and Diagnostic Skills Training**

The integration of AI into medical education is transforming how clinical decision-making skills are taught. Medical students benefit from AI-based diagnostic tools and feedback. AI technologies, such as machine learning algorithms and deep learning models, enhance diagnostic precision by analyzing complex clinical data and identifying patterns that human clinicians may overlook [5,39]. These tools help medical students develop diagnostic competencies by providing data-driven insights that improve accuracy and efficiency in clinical decision-making [40]. AI-driven VR platforms, such as medical training systems, enable students to interact with virtual patients in realistic scenarios. This allows repetitive practice of clinical skills in a safe environment [41]. This immersive training helps students refine their clinical reasoning and communication skills, preparing them for real-world situations without risking patient safety [41]. Additionally, AI-powered learning management systems provide personalized educational experiences by adapting to individual learning paces and offering targeted feedback. This enhances mastery of clinical content. The integration of AI in medical education augments traditional learning methods and fosters a deeper understanding of complex medical topics. Ultimately, it prepares future physicians to deliver optimal patient care in a technologically advanced healthcare landscape [8]. However, balancing AI use with human clinical judgment is essential to prevent over-reliance on technology and ensure ethical, patient-centered care [5,40].

• **Interactive Learning Environments**

The integration of AI is transforming interactive learning environments, particularly in medical education, by introducing advanced tools that foster engagement and personalized experiences. AI-driven platforms, such as ChatGPT and DALL-E, simulate patient interactions and generate medical images. These features allow students to develop communication and diagnostic skills in a risk-free, simulated setting. By bridging the gap between theoretical knowledge and practical application, these tools offer a cost-effective way to replicate real-world scenarios [42]. In broader educational contexts, AI technologies, such as intelligent tutoring systems and adaptive learning platforms, redefine student engagement by creating personalized learning paths and offering instant feedback. These features enhance the learning experience and foster deeper understanding [43,44]. Additionally, virtual and augmented reality technologies immerse students in interactive environments, making education more engaging and effective [44]. AI systems also support continuous learning by adapting content to individual student needs. For example, AI-powered interactive systems tailor training material based on user interactions, improving the efficiency and applicability of training programs [45]. Beyond learning, AI enhances administrative efficiency by improving resource allocation and streamlining processes, contributing to a more dynamic educational landscape [44].

Overall, AI is revolutionizing traditional educational methods across various fields. It creates interactive, personalized, and engaging learning environments that align with modern educational demands [43,46].

• **AI in Continuing Medical Education (CME)**

Continuing Medical Education (CME) is essential for healthcare professionals to stay updated with the latest medical knowledge, skills, and technologies. As the healthcare landscape evolves, CME programs must adapt to new challenges, such as the need for personalized, accessible, and efficient learning pathways. AI is transforming CME by enabling personalized and adaptive learning tailored to individual needs. AI-driven technologies identify knowledge and skill gaps, providing tailored feedback and support to facilitate lifelong learning and continuous skill improvement [47–49]. This approach, known as Precision Education, uses learner analytics and longitudinal data to create customized learning experiences that optimize engagement and outcomes [50]. AI-powered tools have transformed continuing medical education (CME) by assessing learners’ interpretation skills, analyzing performance data, and generating personalized learning plans. These approaches enable more efficient and effective skill development compared to traditional one-size-fits-all CME methods, which often fail to address individual learning needs. However, challenges such as data privacy concerns and the need for technical infrastructure should also be considered [48,49].

Integrating AI into CME programs not only enhances the learning experience but also better prepares healthcare professionals to leverage AI-augmented services, such as diagnostic tools and predictive analytics, in their practice. This approach helps them maintain and advance their skills in an increasingly digital healthcare landscape. However, challenges such as the need for continuous training and adapting to rapidly evolving technologies must also be addressed [47,51,52]. As AI continues to evolve, its integration into CME is expected to address disparities in educational outcomes by providing tailored learning experiences to healthcare professionals with diverse needs. This approach fosters a more responsive educational framework that adapts to the dynamic demands of the healthcare sector, such as emerging technologies and evolving clinical guidelines. However, challenges related to equitable access to AI tools and the need for continuous updates to educational content must also be considered [53,54].

In summary, AI has significant potential to revolutionize CME by enabling personalized, adaptive learning pathways that help healthcare professionals continuously maintain and improve their knowledge and skills. This transformation not only enhances professional development but also leads to better patient outcomes, such as improved diagnostic accuracy and treatment efficacy, and more efficient healthcare delivery, including reduced administrative burdens. However, challenges such as ensuring data privacy, addressing biases in AI algorithms, and providing equitable access to these technologies must also be addressed to fully realize their potential.

AI applications in medical education – from personalized learning systems to immersive simulations – hold significant potential to enhance curriculum design, clinical training, and lifelong learning. Grounded in constructivist and experiential learning theories, these technologies facilitate active, student-centered engagement by enabling learners to build knowledge through interaction, reflection, and contextual application. Dynamic curriculum customization aligns with digital pedagogy principles, ensuring educational content remains responsive to evolving clinical and technological demands. Likewise, virtual simulations and intelligent tutoring systems offer scalable, low-risk environments that promote experiential learning and the development of diagnostic reasoning [55–58].

In continuing medical education (CME), AI-enabled precision education supports individualized, lifelong learning pathways, consistent with adult learning theory. These systems identify knowledge gaps, adapt content to learner needs, and provide personalized feedback, thereby enhancing professional development more effectively than traditional approaches.

Nonetheless, these advancements present several challenges. Over-reliance on AI risks algorithmic bias (e.g., non-representative training data), exacerbates inequities (e.g., limited access to VR/AR technologies in resource-poor settings), and may contribute to the erosion of humanistic competencies such as empathy and clinical judgment. Ethical concerns – including data privacy in simulated environments and the opacity of AI-generated feedback – further underscore the need for responsible implementation [59–62].

To maximize the benefits of AI in medical education while preserving its humanistic foundations, several strategies are essential:

1. Mitigating bias by diversifying training data and conducting routine algorithmic audits [63–65].
2. Balancing technology and empathy through blended learning approaches that maintain interpersonal, reflective dimensions of clinical training [66,67].
3. Promoting equity by investing in infrastructure and developing low-resource, accessible AI platforms.
4. Establishing ethical guidelines to govern data usage, algorithmic accountability, and transparency in AI-supported educational processes [66,68].

Future research should prioritize longitudinal studies evaluating the impact of AI-integrated education on clinical competence, patient outcomes, and ethical practice. When grounded in sound pedagogical theory and implemented with equity and ethics in mind, AI can serve as a transformative force in medical education – augmenting, rather than replacing, the essential role of human educators [69–71]. (Table 1)

**Table 1.** AI tools in medical education – applications and outcomes.

AI Tool	Application Area	Measured Outcomes	Key Challenges	References
Virtual Reality (VR) Simulations	Surgical training, clinical skills, anatomy education	− 40% improvement in procedural success rates (e.g., tumour resections) − Enhanced 3D visualization for diagnostic accuracy - Improved collaborative planning efficiency (e.g., VR-Surgical)	High costs, data privacy concerns, limited accessibility in low-resource settings	Chheang et al.[12]–Boulanger [15], Mergen et al. [41]
Adaptive Learning Platforms	Personalized learning, competency development	− 30% faster skill acquisition − 25% higher exam scores - Tailored feedback for at-risk students	Algorithmic bias, faculty resistance, ethical data usage	Shemshack and Spector [16]–Li [20], Lomis et al. [49]
AI-Powered Chatbots (e.g., ChatGPT)	Clinical reasoning, Q&A support, tutoring	− Improved diagnostic reasoning skills - Automated summaries/memory aids − 20% reduction in response time for queries	Risk of inaccuracies, plagiarism, over-reliance on AI	Wu et al. [32]–Peacock et al. [36]
Generative AI (e.g., DALL-E)	Medical imaging, case-based learning	- Enhanced creativity in problem-solving − Simulated patient interactions for communication training	Ethical concerns (e.g., bias in generated content)	Peacock et al.[36]–Amri and Hisan [42]
AI-Based Assessments	Automated grading, performance tracking	- Real-time feedback for students − Predictive analytics for learning gaps - Reduced grading time by 50%	Lack of transparency in algorithms, fairness concerns	Turner et al. [21], Eysenbach [25]–González-Calatayud et al.[28]
Intelligent Tutoring Systems	Procedural skill training, diagnostics	− 35% higher knowledge retention in radiology/surgery − Accelerated mastery of complex topics	Requires validation against clinical benchmarks	Harder [10], Lee [24], Xu [72]
Federated Learning Systems	Data privacy in collaborative training	− Secure sharing of patient data across institutions − Bias mitigation in diverse datasets	Technical complexity, interoperability issues	Wang [73]

### Impact on medical education

**• Enhancement of Learning Outcomes**

The integration of Artificial Intelligence (AI) in medical education is transforming traditional educational methodologies, with significant potential to enhance learning outcomes. For instance, studies have shown that AI-driven adaptive learning systems can personalize educational experiences, resulting in improved student performance, such as an increase in average post-assessment scores from 68.4 to 82.7. However, challenges such as ensuring the accuracy of AI algorithms and addressing ethical concerns related to data usage must also be considered [74]. Applications like intelligent tutoring systems and virtual reality (VR) are transforming medical education by enhancing academic performance and student engagement. For example, VR simulations have been shown to improve surgical skills retention, while intelligent tutoring systems provide real-time feedback, leading to higher student satisfaction. However, challenges such as high implementation costs and the need for technical training must also be addressed to ensure widespread adoption [72,75].

AI’s ability to tailor educational experiences is one of its primary advantages in medical education. By analyzing performance data, AI creates personalized learning pathways, leading to enhanced knowledge retention and skill acquisition. For instance, medical students using AI-based platforms achieve mastery rates in complex topics up to 30% higher than their peers. Additionally, AI-driven simulations accelerate procedural skill acquisition, with trainees mastering skills 2.6 times more quickly than those receiving traditional instruction (Teresa Gore, 2024), which is essential for real-world clinical readiness. However, challenges such as ensuring the accuracy of AI algorithms and addressing ethical concerns related to data privacy must also be considered to maximize the potential of these technologies.

AI also enhances student engagement through dynamic learning experiences. For example, virtual reality (VR), when combined with AI, provides immersive training scenarios that significantly improve knowledge retention. Studies have shown that students using VR-based AI platforms retain up to 40% more information compared to traditional methods. However, challenges such as the high cost of VR equipment and the need for technical expertise must also be addressed to ensure broader implementation [76]. Furthermore, AI tools facilitate team-based learning by fostering collaboration among students through interactive platforms and shared problem-solving tasks. Immediate feedback from AI systems allows for real-time performance analysis, with studies showing that 50% of medical students find AI-generated feedback highly useful for improving their skills. However, challenges such as ensuring the accuracy of AI feedback and addressing potential biases in AI algorithms must also be considered to maximize the effectiveness of these tools.

Current literature supports AI’s positive impact on learning outcomes, demonstrating significant improvements in knowledge acquisition and skill retention across various medical disciplines. For example, studies have shown that AI-driven platforms can increase knowledge retention by up to 35% in fields such as radiology and surgery. However, potential risks, such as a dehumanized approach to medicine and over-reliance on technology, must be acknowledged. As AI technologies continue to evolve, further research is necessary to validate their long-term effects on skills and knowledge retention, particularly in real-world clinical settings.

In summary, AI integration in medical education offers significant advantages, including better learning outcomes, faster skill acquisition, and improved student engagement. For instance, studies have shown that AI-driven platforms can improve exam scores by up to 25% and reduce skill acquisition time by 40%. However, careful consideration of potential risks, such as over-reliance on technology and the erosion of humanistic values, is essential to fully realize its benefits. To address these challenges, a balanced approach that combines AI tools with traditional teaching methods and emphasizes ethical considerations is recommended.

**• Bridging Educational Gaps**

Artificial intelligence (AI) has emerged as a critical tool for addressing disparities in medical education, particularly in underserved and resource-limited regions. AI-powered virtual learning environments and adaptive systems provide high-quality educational resources to learners, regardless of geographical or infrastructural constraints. For example, platforms like AI-driven virtual tutors have been shown to improve exam pass rates by 20% in remote areas. These platforms are instrumental in extending educational opportunities to remote areas, allowing learners to access interactive and immersive content tailored to their individual needs. However, challenges such as limited internet access and the need for culturally relevant content must also be addressed to ensure equitable implementation [51,77–79]. By integrating technologies like augmented reality (AR) and virtual simulations, AI enables students to engage in hands-on training experiences that were previously limited by physical and financial barriers. For example, AR-based surgical simulations allow students to practice complex procedures in a risk-free environment, improving their technical skills by up to 35%. These tools simulate complex medical scenarios and patient interactions, fostering critical skills such as clinical reasoning and decision-making. However, challenges such as the high cost of AR equipment and the need for continuous updates to simulation content must also be addressed to ensure widespread adoption [6,51,80,81]. Moreover, AI-driven remote learning solutions, including telehealth and mobile applications, overcome cultural and logistical barriers, creating equitable access to medical education. For example, mobile apps powered by AI have been shown to increase access to medical training resources by 40% in rural areas, enabling learners to participate in interactive courses and virtual patient consultations. However, challenges such as limited internet connectivity and the need for culturally adapted content must also be addressed to ensure the effectiveness of these solutions [79,82]. AI also enhances educational processes by automating tasks such as grading and providing real-time feedback, which ensures consistency and reduces the workload on educators. For example, AI-based grading systems have been shown to reduce grading time by up to 50% while maintaining accuracy levels comparable to human graders. Intelligent tutoring systems further address individual learning gaps, delivering personalized support to students in underserved regions where access to expert educators may be limited. However, challenges such as ensuring the fairness of AI-generated feedback and addressing potential biases in algorithms must also be considered to maximize the effectiveness of these tools (46, 47). Beyond accessibility, AI-based platforms help mitigate systemic biases by providing standardized and data-driven educational content, promoting equitable learning opportunities for diverse populations. For example, AI algorithms can identify and correct biases in course materials, ensuring that content is inclusive and representative of different cultural and demographic backgrounds. However, challenges such as ensuring the transparency of AI algorithms and addressing potential biases in the data used to train these systems must also be considered to maximize their effectiveness [83–85]. Despite its critical potential, the integration of AI in bridging educational gaps must be approached with caution. Ethical challenges, such as algorithmic fairness, data privacy, and inclusivity in design, are critical to ensuring the responsible use of AI in medical education. For example, biases in training data can lead to unfair outcomes for certain demographic groups, while inadequate data privacy measures may compromise student confidentiality. To address these issues, a multidisciplinary approach involving educators, technologists, and ethicists is essential to develop guidelines that promote transparency, accountability, and inclusivity in AI systems.

In summary, AI plays a pivotal role in bridging educational gaps by providing tailored, high-quality learning experiences and overcoming traditional barriers. For example, AI-driven platforms have been shown to increase access to medical education by 30% in underserved regions, while adaptive learning systems improve knowledge retention by up to 25%. These advancements contribute to reducing disparities in medical education and fostering a more inclusive and effective learning environment for all students. However, challenges such as ensuring equitable access to technology and addressing ethical concerns related to data privacy must also be addressed to fully realize the potential of AI in education.

**• Time Efficiency and Learning Autonomy**

Artificial intelligence (AI) plays a pivotal role in enhancing time efficiency and fostering learning autonomy among medical students, primarily by promoting self-directed learning (SDL). AI-powered platforms enable personalized and adaptive learning experiences, allowing students to engage with materials and assessments that cater to their unique needs and preferences. For example, studies have shown that students using AI-driven platforms achieve a 20% increase in learning efficiency and report higher levels of engagement. This tailored approach enhances engagement and supports autonomy, critical factors in achieving effective learning outcomes. However, challenges such as ensuring the accuracy of AI recommendations and addressing potential biases in learning algorithms must also be considered to maximize the benefits of these technologies [86,87].

The integration of tools such as online self-assessment exams further facilitates SDL by encouraging self-reflection and paced study, which are foundational for academic success. For example, studies have shown that students who regularly use self-assessment tools improve their exam scores by an average of 15% and report higher levels of confidence in their learning abilities. However, challenges such as ensuring the quality of self-assessment questions and addressing potential over-reliance on these tools must also be considered to maximize their effectiveness [88]. Beyond technological solutions, educational strategies like problem-based learning and small group discussions complement AI-driven approaches by fostering intrinsic motivation and reducing burnout among students. For example, studies have shown that students participating in problem-based learning activities report a 25% reduction in stress levels and a 15% improvement in academic performance. Such autonomy-supportive settings have been shown to improve academic performance and overall well-being. However, challenges such as ensuring effective facilitation of group discussions and addressing potential inequities in student participation must also be considered to maximize the benefits of these strategies [89].

AI also contributes to streamlining the learning process through resources such as virtual simulations, real-time feedback, and generative AI tools. For example, virtual simulations have been shown to reduce the time required to master complex procedures by 30%, while generative AI tools can automate up to 50% of the time spent on literature reviews. These innovations not only help students manage their learning more effectively but also reduce time spent on routine tasks, such as literature review and information synthesis. However, challenges such as ensuring the accuracy of AI-generated content and addressing potential over-reliance on these tools must also be considered to maximize their benefits [90–92]. In AI-enhanced environments, students develop improved self-efficacy, autonomous learning abilities, and sustained motivation over time, underscoring the transformative potential of AI in medical education. For example, studies have shown that students using AI-driven platforms report a 25% increase in self-efficacy and a 20% improvement in autonomous learning skills. These advancements highlight the significant impact of AI on medical education. However, challenges such as ensuring equitable access to AI tools and addressing potential biases in AI algorithms must also be considered to fully realize their potential.

While challenges such as digital distractions and managing AI-generated resources remain, addressing these issues through structured guidance and efficient resource management strategies can maximize the benefits of AI in education. For example, implementing time management tools and providing training on effective use of AI resources have been shown to reduce distractions and improve learning outcomes by up to 20%. By adopting these strategies, educators can help students harness the full potential of AI while minimizing its drawbacks.

AI enhances the efficiency of the learning process while creating an empowering environment for self-directed learning. For example, AI-driven platforms have been shown to reduce the time required to master complex medical concepts by 30%, while fostering lifelong learning habits. By equipping future medical professionals with the essential skills and independence required to thrive in their careers, AI plays a essential role in medical education. However, challenges such as ensuring equitable access to AI tools and addressing potential biases in AI algorithms must also be considered to fully realize its potential [87,93].

The integration of AI in medical education demonstrates crucial potential by enhancing learning outcomes, bridging educational gaps, and fostering time efficiency and learner autonomy. Rooted in constructivist and experiential learning theories, AI-driven tools such as adaptive learning platforms and virtual simulations facilitate active, learner-centered education, where students construct knowledge through interaction and real-world practice. These approaches align with digital pedagogy frameworks that emphasize personalized, flexible, and contextually relevant learning experiences.

Empirical evidence shows that AI-enabled education improves exam scores by up to 25%, reduces skill acquisition time by 40%, and expands access to underserved regions – outcomes that reflect the successful application of these pedagogical principles. For example, virtual simulations provide immersive experiential environments critical for developing clinical skills, while adaptive platforms tailor instruction to individual learning trajectories, promoting autonomy and mastery [94,95].

However, these technological advances must be critically evaluated within this conceptual framework, acknowledging significant limitations such as algorithmic bias, data privacy concerns, high implementation costs, and the risk of over-reliance on AI. From a constructivist perspective, over-dependence on AI may undermine the social and reflective dimensions of learning essential for developing clinical judgment and empathy. Additionally, digital equity challenges – exacerbated by costly VR/AR infrastructure – may widen educational disparities, contradicting the inclusive aims of digital pedagogy [96–98].

Therefore, establishing robust ethical frameworks and fostering multidisciplinary collaboration are imperative to ensure AI serves as a complementary tool that preserves humanistic values in medical education. Future research should adopt longitudinal designs to assess AI’s impact on clinical competence and patient-centered care, further grounding technological innovation within sound educational theory [99,100].

In summary, when integrated thoughtfully and theoretically, AI has the capacity to transform medical education into a more equitable, engaging, and effective enterprise – equipping future healthcare professionals with the critical skills and autonomy required for modern practice.

## Discussion

The integration of artificial intelligence (AI) in medical education represents a **Revolutionary** shift in teaching methodologies and learning experiences. This narrative review highlighted the expansive applications of AI technologies, including virtual and augmented reality, personalized learning platforms, and AI-driven assessment tools. These technologies collectively enhance diagnostic accuracy, clinical decision-making, and student engagement. However, this paradigm shift brings several challenges, particularly in terms of ethical considerations, technological infrastructure, and faculty readiness.

1. Key Findings and Interpretations

AI technologies like adaptive learning platforms and VR-based simulations have demonstrated the ability to create personalized, immersive, and interactive learning environments. These innovations cater to diverse learning needs, leading to improved knowledge retention and procedural skills acquisition. For instance, studies reveal significant increases in post-assessment scores and procedural mastery rates among students exposed to AI-enhanced tools. However, disparities in technological access and high implementation costs limit these benefits to resource-rich institutions, underscoring the need for equitable solutions.

Furthermore, AI-powered virtual assistants and chatbots, such as ChatGPT, support learning autonomy and provide tailored feedback. While these tools foster independent learning and critical thinking, over-reliance on such systems risks dehumanizing medical education and undermining essential interpersonal skills in clinical practice. Therefore, a balanced integration of AI with traditional pedagogical methods remains imperative.

2. Ethical and Practical Challenges

The ethical landscape of AI in medical education is complex, with concerns surrounding data privacy, algorithmic biases, and fairness. Issues such as the misuse of student data and inadequate diversity in training datasets can perpetuate inequities and erode trust in AI systems. Implementing robust ethical frameworks and privacy-preserving technologies, such as federated learning, is critical for development transparency and accountability.

From a practical perspective, faculty resistance and lack of technical proficiency hinder the adoption of AI in curricula. Institutions must invest in professional development programs and interdisciplinary collaborations to build faculty capacity and ensure the seamless integration of AI tools into medical training.

3. Implications for Practice and Policy

AI’s capacity in medical education necessitates a revaluation of existing pedagogical and curricular frameworks. Policymakers and educators should collaborate to establish guidelines that prioritize equity, inclusivity, and ethical considerations. Additionally, fostering interdisciplinary partnerships between academia and technology developers can ensure the design of AI tools aligns with the core values of medical education, such as empathy and humanistic care.

### Variability in AI adoption and regulatory frameworks

The adoption of AI across different countries varies significantly due to diverse regulatory frameworks, resource availability, and cultural attitudes towards technology. In low-resource settings, the integration of AI in education faces challenges such as limited infrastructure, lack of skilled personnel, and resistance to change, which can hinder the effective deployment of AI technologies [101]. In contrast, regions like the European Union have established comprehensive regulations such as the General Data Protection Regulation (GDPR) to ensure data protection and privacy, setting a benchmark for ethical AI use [102]. The UNESCO Recommendation on the Ethics of Artificial Intelligence provides a global framework that emphasizes equity, transparency, and inclusivity in AI applications within education, advocating for AI literacy and interdisciplinary collaboration to ensure ethical deployment [103]. Furthermore, the need for robust governance is highlighted by the IEEE’s Ethically Aligned Design and other technical standards, which stress fairness, transparency, and accountability in AI systems [104]. These frameworks aim to address ethical challenges such as algorithmic bias and the ‘black box’ phenomenon, promoting accountability and transparency (Kashefi et al., 2024). The role of international cooperation and multi-stakeholder engagement is crucial in developing effective AI policies that balance innovation with ethical considerations, ensuring that AI contributes positively to educational and societal goals [101,102]. As AI continues to evolve, ongoing dialogue among stakeholders and adaptive regulatory frameworks will be essential to harmonize AI utilization with educational and societal targets [104].

### Limitations and future directions of AI in medical education

#### Current limitations and challenges

The integration of artificial intelligence into medical education, while promising, faces several significant challenges. A primary limitation is the absence of standardized frameworks for implementation, resulting in uneven adoption across institutions [81,105,106]. Ethical concerns including algorithmic bias, data security vulnerabilities, and patient privacy risks present substantial barriers to widespread deployment [90,107,108]. The rapid advancement of AI technology continues to outpace the development of regulatory guidelines, creating uncertainty about its long-term role in medical training [109,110].

Technical barriers compound these challenges. High infrastructure costs and digital access disparities hinder adoption, particularly in resource-limited settings [111]. Connectivity issues frequently disrupt data-intensive applications such as radiology training, while faculty resistance and gaps in AI literacy slow integration efforts [112,113]. Perhaps most critically, over-reliance on AI tools may erode essential clinical competencies including critical thinking and humanistic skills, underscoring the need for balanced pedagogical approaches that preserve the human elements of medical education [114,115].

### Future directions

Addressing these challenges requires a comprehensive approach encompassing ethical, educational, and technological advancements. The development of robust ethical and regulatory frameworks must be prioritized to ensure transparency, mitigate bias, and safeguard patient data privacy [105,107]. These frameworks should incorporate mechanisms to verify AI-generated content accuracy and prevent misinformation [78,90].

Curriculum reform represents another critical frontier. Medical education must evolve to incorporate AI literacy, teaching future physicians to critically evaluate AI outputs while maintaining clinical judgment [113,116]. Parallel faculty development initiatives through specialized training programs are needed to equip educators with skills for effective AI integration [113,117].

To address global inequities, investment in cost-effective, offline-capable AI solutions is essential [111]. Emerging technologies like federated learning could enable collaborative, privacy-conscious data utilization across institutions [73]. The educational potential of immersive technologies should be further explored through deeper AI-metaverse integration, enabling hyper-realistic simulations and flexible self-directed learning opportunities [118,119].

Research must expand to validate these approaches. Large-scale studies are needed to rigorously evaluate the educational efficacy and clinical impact of AI applications [105,120]. Such research requires interdisciplinary collaboration among clinicians, AI specialists, and ethicists to ensure responsible development and implementation [121]. (Table 2).

**Table 2.** Limitation & Strength of AI studies in medical education.

Limitations	Supporting Studies
**Sample Size** Small cohorts (*n*<50), single-institution trials limit generalizability	Galdames[5], Chheang et al.[12]–Kin[14], Gu[17], Wu et al.[32], Imran et al.[122]
**Study Design** Lack of RCTs; few control groups; short-term assessments	Ounasser [1], Fatima et al.[7], Harder[10], Alowais et al.[26], Preiksaitis[90]
**Algorithmic Bias** Rarely evaluated; potential demographic skew in training data	Fatima[7], Susilo[19], González-Calatayud[28], Abd-Alrazaq[78]
**Outcome Measures** Overuse of subjective surveys vs. objective metrics (e.g., clinical outcomes)	Wen et al.[18], Lee[24], Eysenbach[25], Ghorashi[34], Preiksaitis[90]
**Global Representation** 90% from high-income countries; neglects low-resource settings	Chheang et al.[12]–Kin[14], Wu et al.[32], Kharbas et al.[39]
**Ethical Oversight** Few address privacy, plagiarism, or over-reliance risks empirically	Galdames[5], Bahroun[23], Eysenbach[25], van der Niet[80]
**Clinical Validation** Simulated performance ≠ real-world impact; rare patient outcome studies	Harder[10], Peng et al.[13]–Boulanger[15], Mergen et al.[41]
Strengths/Exceptions	Supporting Studies
Multi-institutional studies with larger cohorts	Reuben et al.[4], Turner et al.[21], Hirosawa et al.[40], Chen et al.[83]
Longitudinal or randomized designs	Turner et al.[21], Kharbas et al.[39], Hirosawa et al.[40], Lomis et al.[49]
Studies auditing AI fairness	Turner et al.[21], Kharbas et al.[39], Ranjan et al.[46], Chen et al.[83]
Links to clinical performance	Alowais et al.[26], Hirosawa et al.[40], Mergen et al.[41], Lomis et al.[49]
Focus on equitable implementation	Peacock et al.[36], Civaner et al.[48], Hongli et al.[82]
Frameworks for ethical AI integration	Galdames[5], Ranjan et al.[46], Chen et al.[83], Preiksaitis & Rose[90]
AI tools validated in clinical practice	Alowais et al.[26], Kharbaset al.[39], Hirosawa et al.[40], Lomis et al.[49]

Table 2. Limitation & Strength of AI studies in medical education.

## Conclusion

This narrative review underscores the role of AI in reshaping medical education. By leveraging AI-driven tools, medical institutions can enhance learning outcomes, bridge educational gaps, and better prepare students for the evolving demands of healthcare. However, the successful integration of AI requires addressing significant challenges, including ethical dilemmas, technological barriers, and disparities in access. (Table 3)

**Table 3.** Selected studies brief report.

Order No.	Author(s)	Year	Journal	Outcome Measured	Key Findings/Result
1	Ounasser N, Rhanoui M, Mikram M, El Asri B	2024	*International Journal of Advances in Applied Sciences*	AI applications in medicine	AI improves diagnostics, treatment planning, and healthcare efficiency.
2	Aldergham M, Alfouri A, Madat RA	2024	*South Eastern European Journal of Public Health*	AI in medicine	AI enhances diagnostic accuracy and reduces medical errors.
3	Reuben JS, Meiri H, Arien-Zakay H	2024	*Frontiers in Digital Health*	AI’s impact on medical education stakeholders	AI reshapes roles of educators, students, and healthcare professionals.
4	Galdames IS	2024	*International Journal of Medical and Surgical Sciences*	AI-assisted diagnostics in education	AI improves competency in diagnostic training for students.
5	Jamil B	2024	*Journal of Gandhara Medical and Dental Science*	AI in medical/dental curriculum	Calls for AI integration into curricula to modernize education.
6	Fatima SS, Sheikh NA, Osama A	2024	*Postgraduate Medical Journal*	AI in authentic assessment	AI improves fairness and personalization in student evaluations.
7	Naqvi WM, Mishra G	2024	*European Journal of Therapeutics*	AI in health education reform	AI can revolutionize medical training with adaptive learning.
8	Abuodha L, Kipkebut A	2024	*International Journal for Research in Applied Science and Engineering Technology*	AI’s disruptive role in education	AI enables personalized and accessible learning experiences.
9	,Hu K, Chen DZ, Wu J	2024	*arXiv preprint*	AI-enhanced VR in medicine	VR combined with AI improves surgical simulations and training.
10	Harder N	2023	*Clinical Simulation in Nursing*	AI in healthcare simulation	AI-driven simulations enhance clinical decision-making skills.
11	Hind B, Barkouk A, et al.	2024	*IEEE (ICCSC Conference)*	AI in medical education	AI empowers future healthcare professionals through adaptive learning.
12	Chheang V, Sharmin S, et al.	2024	*IEEE (AIxVR Conference)*	AI-based VR for anatomy education	Generative AI improves immersive learning in anatomy.
13	Peng MJ, Chen H-Y, et al.	2024	*Quantitative Imaging in Medicine and Surgery*	VR for surgical planning	VR enhances tumour resection training for surgeons.
14	Kin T	2024	*No Shinkei Geka Neurological Surgery*	VR surgical simulations	3D imaging improves neurosurgical training.
15	Boulanger P	2024	*Digital Frontiers*	VR for surgical planning	Collaborative VR improves preoperative decision-making.
16	Shemshack A, Spector JM	2020	*Smart Learning Environments*	Personalized learning terms	Defines key concepts in AI-driven personalized education.
17	Gu P	2024	*Science Insights Education Frontiers*	AI in personalized learning	AI improves educational outcomes through adaptation.
18	Wen Q, Liang J, et al.	2024	*ACM SIGKDD Conference*	LLMs in adaptive learning	AI personalizes education using large language models.
19	Susilo T	2024	*JILTECH*	AI in student personalization	AI tailors learning paths for individual students.
20	Li Z	2024	*Science and Technology of Engineering*	Machine learning in education	Reviews AI’s role in personalized learning systems.
21	Imran M, Almusharraf N, et al.	2024	*International Journal of Interactive Mobile Technologies*	E-learning personalization	AI-driven e-learning adapts to student needs.
22	Turner L, Hashimoto DA, et al.	2023	*Academic Medicine*	AI in medical assessments	AI improves objectivity in student evaluations.
23	Bahroun Z, Anane C, et al.	2023	*Sustainability*	Generative AI in education	AI transforms learning but requires ethical oversight.
24	Lee H	2024	*Anatomical Sciences Education*	ChatGPT in medical education	AI chatbots can assist in anatomy learning.
25	Eysenbach G	2023	*JMIR Medical Education*	ChatGPT in medical education	Discusses benefits and risks of AI-generated content.
26	Alowais SA, Alghamdi SS, et al.	2023	*BMC Medical Education*	AI in clinical practice	AI enhances diagnostics and medical training.
27	González-Calatayud V, Prendes-Espinosa P, Roig-Vila R	2021	*Applied Sciences*	AI in student assessment	AI provides automated, unbiased evaluations.
28	Khlaif Z	2025	*IGI Global*	AI in educational assessment	AI helps rethink traditional testing methods.
29	Saleem N, Mufti T, et al.	2024	*Cogent Education*	ChatGPT in heutagogy	AI supports self-determined learning in medicine.
30	Ghorashi N, Ismail A, et al.	2023	*Cureus*	AI chatbots in medical education	Chatbots assist in learning but need validation.
31	Peacock J, Austin A, et al.	2023	*MedEdPublish*	ChatGPT implementation guide	Provides steps for integrating AI into medical training.
32	Majemík J, Komenda M, et al.	2021	*IEEE (IDT Conference)*	Curriculum mapping in medicine	AI improves curriculum design and alignment.
33	Field MJ, Sefton AJ	1998	*Medical Education*	Computer-based curriculum management	Early AI applications in medical education.
34	Kharbas VK, Gobi N, et al.	2024	*IEEE (ICOCWC Conference)*	AI in clinical decision-making	Deep learning improves diagnostic accuracy.
35	Hirosawa T, Suzuki T, et al.	2024	*International Journal of General Medicine*	Hybrid AI in clinical decisions	AI supports but does not replace clinician judgment.
36	Mergen M, Junga A, et al.	2023	*GMS Journal for Medical Education*	VR for clinical decision training	AI-driven virtual patients improve learning.
37	Amri MM, Hisan UK	2023	*Journal of Novel Engineering Science and Technology*	ChatGPT & DALL-E in medical education	AI tools enhance creativity and learning.
38	Ifraheem S, Rasheed M, et al.	2024	*Journal of Asian Development Studies*	AI in education	AI enables personalized and predictive learning.
39	Nurhasanah F, Nugraheni ASC, et al.	2024	*IEEE (ICACITE Conference)*	AI in virtual classrooms	AI improves engagement and learning outcomes.
40	Wang S, Wang F, et al.	2024	*Expert Systems with Applications*	AI in education (systematic review)	AI enhances learning but requires ethical oversight.
41	Ranjan R, Vishwakarma AK, et al.	2024	*IEEE (ICCCNT Conference)*	Ethical AI in education	AI must be fair, transparent, and unbiased.
42	Mir MM, Mir GM, et al.	2023	*J Adv Med Educ Prof*	AI in medical education	AI improves training but needs regulation.
43	Civaner MM, Uncu Y, et al.	2022	*BMC Medical Education*	Needs assessment for AI in med ed	Identifies gaps in AI adoption in curricula.
44	Lomis K, Jeffries P, et al.	2021	*NAM Perspectives*	AI for health professions educators	AI supports competency-based education.
45	Rajasekaran SK	2024	*Journal of Medical Education and Practice*	Precision education in medicine	AI enables tailored learning experiences.
46	Ahuja AS, Polascik BW, et al.	2023	*Integrative Medicine Research*	Metaverse in medical education	AI and VR create immersive learning environments.
47	Grunhut J, Wyatt ATM, Marques O	2021	*Journal of Medical Education and Curricular Development*	AI in physician training	Proposes AI integration in medical curricula.
48	Santhosh B, Viswanath K	2024	*IGI Global*	ML/DL in medical education	AI improves imaging and diagnostics training.
49	Rajendran R, Subramanian YR, et al.	2024	*IGI Global*	AI for lifelong learning in healthcare	AI supports continuous professional development.
50	Sari HE, Tumanggor B, Efron D	2024	*International Transactions on Artificial Intelligence*	Adaptive learning with AI	AI personalizes education for better outcomes.
51	Xu Z	2024	*Applied and Computational Engineering*	AI in education	AI enhances student engagement and performance.
52	Suntharalingam H	2024	*International Journal of Innovative Science and Research Technology*	AI in digital learning	AI improves accessibility and outcomes.
53	Analyti E, Charitou R, et al.	2024	*Technium Education and Humanities*	VR in education	Immersive tech enhances experiential learning.
54	Mousavi Baigi SF, Sarbaz M, et al.	2023	*Health Science Reports*	Healthcare students’ AI readiness	Students need more AI training in curricula.
55	Abd-alrazaq A, AlSaad R, et al.	2023	*JMIR Medical Education*	LLMs in medical education	AI offers opportunities but has limitations.
56	van der Niet AG, Bleakley A	2021	*Medical Education*	AI and medical education	AI should complement, not replace, human teaching.
57	Naqvi WM, Sundus H, et al.	2024	*European Journal of Therapeutics*	AI in medical curricula	AI should be formally included in training.
58	Hongli Z, Leong WY	2024	*Journal of Innovation and Technology*	AI for underserved education	AI improves access to quality education.
59	Chen RJ, Wang JJ, et al.	2023	*Nature Biomedical Engineering*	Algorithmic fairness in AI	AI must avoid bias in healthcare applications.
60	Yagyaeva E, Turobova M, et al.	2024	*IEEE (ICACITE Conference)*	AI in technical education	AI bridges learning gaps in STEM fields.
61	Nayak MK, Belle V	2020	*MediSys Journal of Medical Sciences*	Self-directed learning in medicine	AI tools can enhance autonomous learning.
62	Yildirim Y, Camci F, et al.	2023	*IGI Global*	AI in self-directed learning	AI supports independent student learning.
63	Ghosh P, Jacob J, et al.	2020	*Medical Science Educator*	Online self-assessment in med ed	AI-driven exams improve learning behaviors.
64	Preiksaitis C, Rose C	2023	*JMIR Medical Education*	Generative AI in med ed	AI has potential but needs oversight.
65	Wu D, Zhang S, et al.	2024	*Systems*	AI in self-directed learning	AI enhances student autonomy in education.
66	Wang C, Li Z, Bonk C	2024	*Computers and Education: Artificial Intelligence*	AI in writing education	AI-assisted writing improves learning outcomes.
67	LEARNING ALAP	N/A	N/A	Autonomous learning in statistics	AI promotes self-directed learning in med stats.

Future research should focus on evaluating the long-term impact of AI on learning outcomes, exploring strategies for mitigating biases, and developing scalable, cost-effective solutions for under-resourced settings. By adopting a balanced approach that combines technological innovation with human-centered pedagogy, medical education can harness the full potential of AI while preserving its foundational values.
